# Early Growth Performance of In Vitro Raised *Melia volkensii* Gürke Plantlets in Response to Beneficial Microorganisms under Semi-Arid Conditions

**DOI:** 10.3390/plants11101300

**Published:** 2022-05-13

**Authors:** Constantin Dushimimana, Michael Ajanja Sakha, Mercy Jebiwott Korir, Joyce Mnyazi Jefwa, Jan Vandenabeele, Titus Magomere, Eunice Wanjiru Mutitu, Jackson Mulatya, Florence Olubayo, Guy Smagghe, Stefaan P. O. Werbrouck

**Affiliations:** 1 Department of Plants and Crops, Faculty of Bioscience Engineering, Ghent University, Coupure Links 653 and Valentin Vaerwyckweg 1, B-9000 Ghent, Belgium; guy.smagghe@ugent.be; 2 Department of Plant Science and Crop Protection, University of Nairobi, Kangemi, Nairobi P.O. Box 29053-00625, Kenya; magomere.titus@ku.ac.ke (T.M.); mutitu@uonbi.ac.ke (E.W.M.); olubayo@uonbi.ac.ke (F.O.); 3 Mycology Laboratory, Botany Department, National Museums of Kenya, Nairobi P.O. Box 40658-00100, Kenya; sirher.sakha225@gmail.com (M.A.S.); mercykorir3317@gmail.com (M.J.K.); joycejefwa@gmail.com (J.M.J.); 4 Better Globe Forestry, Nairobi P.O. Box 823-00606, Kenya; jan@betterglobeforestry.com; 5 Kenya Forestry Research Institute, Nairobi P.O. Box 20412-00200, Kenya; jmulatya@kefri.org

**Keywords:** *Melia volkensii*, biological agents, micropropagation, acclimatization, field, arid and semi-arid areas

## Abstract

Before in vitro propagated *Melia volkensii* plants can be used for mass planting, the transition phase to in vivo conditions needs to be better controlled because too many plants are lost during acclimatization and in the field. Two experiments were set up to evaluate the effects of biological agents on the establishment of *M. volkensii* in vitro plantlets. The biological agents consisted of Trichotech^®^, Bio-cure B^®^, Rhizatech^®^, *Bacillus subtilis,* a *Trichoderma* isolate and self-isolated native arbuscular mycorrhizal fungi (AMF). Regarding the latter, in soil from the nursery, the number of AMF spores increased from six spores to 400 per 100 g of soil using a trap culture, in which thirteen AMF morphotypes were identified and root colonization assessed through observation of hyphae, vesicles, coils and appressoria. The first experiment was set up in the greenhouse to investigate the efficacy of the biological agents on the hardening off. In the second, a field experiment was set up to study their effect on the early establishment of the plantlets in the field compared to seedlings. All biological agents significantly (*p* ≤ 0.05) improved in vitro plant survival and growth compared to the control. The highest plant height and number of leaves per plant were recorded in plants treated with Rhizatech^®^, Native AMF, Bio-cure B^®^ and *Trichoderma* isolate. The treatments with Rhizatech^®^, Bio-cure B^®^ and native mycorrhiza recorded a significantly wider stem. The root diameter of the plants treated with Rhizatech^®^ and Bio-cure B^®^ was the largest, but the plants inoculated with the native AMF had the longest roots. Moreover, the inoculated plants generally developed multiple secondary roots. After two months, AMF had clearly colonized the acclimatized plantlets. In the field experiment, the biologicals made no difference in survival rate but did produce a significantly larger leaf area after two months, with the largest leaves recorded with Rhizatech^®^, native AMF and Trichotech^®^. They also increased the quality index of the plants from 0.21 to 0.52. The performance of in vitro grown *M. volkensii* plants six months after planting in semi-arid conditions in Kiambere was better than that of seedlings. Inoculation of plants increased plant height and diameter. Thus, inoculation of biological agents is an efficient approach for improving the early growth of in vitro propagated *M. volkensii* plants.

## 1. Introduction

Plant tissue culture plays an increasing role in the propagation, conservation and breeding of tree species [1]. The micropropagation of plantation trees aims to transfer massive numbers of cloned elite plants to the field at a low cost and with as little plant loss as possible. As a consequence of the heterotrophic growth in vitro, the plantlets’ morphology, anatomy, and physiology are suboptimal, requiring much adaptation to survive under harsh environmental conditions. Various techniques such as decreasing the sugar concentration in the medium, increasing light intensity and improving aeration are recommended to prepare in vitro plantlets for external conditions [2]. Moreover, the in vitro plants are grown in sterile conditions and during acclimatization, they have to reach equilibrium with the microbial life again [3]. Different biotic and abiotic stresses, including pests and diseases, low humidity and excess light, have been reported to affect in vitro plantlets during acclimatization [4]. However, for most tree species, including *Melia volkensii* (Mukau), optimal procedures for transferring acclimatized in vitro plantlets to the field have not yet been developed.

*Melia volkensii* is a drought-tolerant [5] versatile tree species from East Africa’s arid and semi-arid areas. Micropropagation is an alternative to seed propagation that is challenging due to harrowing seed extraction and low germination rates [6]. To date, the tissue culture of *M. volkensii* has been primarily limited to the regeneration of shoots [6,7,8,9]. But many other challenges exist, such as inadequate root systems and pathogens attacks, which are the main cause of high mortality and poor growth of *M. volkensii* plantlets after acclimatization. Methods of transferring plantlets from in vitro to the greenhouse for hardening and then the greenhouse to the field for the establishment would be the most outstanding achievement of its micropropagation process.

The use of beneficial microorganisms during acclimatization has been reported to reduce the mortality of in vitro plantlets transferred to the greenhouse [10,11,12,13,14]. In the semi-arid savanna’s soil, *M. volkensii* is significantly associated with five genera of arbuscular mycorrhizal fungi (AMF): these are *Acaulaspora*, *Glomus*, *Gigaspora*, *Scuttelospora* and *Entrophospora* [15]. To date, there is a scarcity of knowledge on the symbiotic effects of different beneficial microorganisms in the course of hardening and the establishment of *M. volkensii* plantlets. Therefore, this study aimed to investigate the influence of commercially available and local microorganisms in this respect.

## 2. Materials and Methods

### 2.1. Plant Material

Fruits from the selected trees were obtained from Better Globe Forestry (BGF), Kenya. After removing the pulp, the nut was cracked open and seeds with an intact seed coat were chosen. They were surface sterilized by rinsing in ethanol 70% and incubating in a 20% JIK^®^ commercial bleach (3.5% *m*/*v* sodium hypochlorite) containing 0.005% detergent (Teepol, Orpington, UK) for 15 min. After the seed coat was cut lengthwise for scarification, they were transferred to test tubes containing Murashige and Skoog’s (MS) medium [16] supplemented with 30 g.L^−1^ sucrose and 2 g.L^−1^ gelrite. The pH of the medium was adjusted to 5.8 before autoclaving at 121 °C for 15 min. Two weeks after germination, the seedlings were divided into nodes to begin micropropagation on the same basal medium supplemented with 5 µM meta-Topolin riboside (mTR). Each subculture lasted four weeks. The best-growing seedling (code 19016) was retained and further subcultured.

The shoots were rooted using a modified McCown woody plant medium [17] with half concentrations of K_2_SO_4_ and MgSO_4_ and supplemented with 3% sucrose, 2 g.L^−1^ gelrite, 0.02 M silver thiosulfate (STS) and 2 µM Indole-3-butyric acid (IBA). Four weeks old in vitro rooted shoots were removed from the media and washed gently with tap water. Then, shoots were transplanted into 300 mL pots containing peat moss (KEKKILA LSM 2 W R8284). To maintain high air humidity, transparent pots were used to cover pots with plants for 12 days. 

### 2.2. Greenhouse and Field Conditions

This study was conducted in both greenhouse and field conditions. The field experiment was performed at Kiambere, BGF station, which is located at 0°41′35.27′′ S latitude and 37°54′56.86′′ E longitude, at an altitude of 722 m above sea level. The experimental field had not been cultivated for more than 30 years. It is located in a semi-arid area with an average annual precipitation of 800 mm. During the experimental period in November 2020, a maximum monthly total rainfall of 252 mm was recorded, while there was no rain at all in March and May 2021 (Table 1). The chemical and physical properties of the soil are presented in Table 2. The soil is classified as sandy clay, slightly acidic (pH = 6.35) and low in organic carbon and total nitrogen.

### 2.3. Indigenous Arbuscular Mycorrhiza Fungi

The soil samples with indigenous AMF were obtained from BGF’s nursery in Kiambere, from pots in which *M. volkensii* seedlings had been grown. They were mixed to obtain a composite soil used for AMF bulking. This was a five-month trap culture experiment and leeks were used as a trap plant. In the last month, watering was decreased to allow AMF sporulation of fungal species present in the vegetative state. The initial and the trap culture soil were assessed as follows. The soil was thoroughly mixed and then AMF spores were isolated and counted from three samples of 100 g each. These were suspended in 200 mL water following the wet sieving and decanting method [18]. The soil was shaken vigorously; the mixture was decanted through 710 and 45 μm mesh sieves. The sievings were distributed into 100 mL tubes containing 25 mL of water. Then, the tubes were shaken well before centrifuging the mixture at 2700 rpm for five minutes. The supernatants were poured out of the tubes while the sediment remained at the bottom of the tubes. A 50% (vol/vol) sucrose solution was added to the tubes to the 30 mL mark before centrifugation at 2700 rpm for one minute. The supernatant at this point was washed through a 45 µm sieve to remove sucrose. The spores were collected in clean water into a 50 mL beaker and were isolated under a dissecting microscope (Olympus SZ-STS). They were transferred to microscopic slides using forceps and mounted in Polyvinyl Alcohol, Lactic acid, Glycerol (PVLG) [19] and a combination of PVLG and Melzer’s reagent (1:1) [20]. Spores were identified under a compound microscope (Olympus CX21) using original descriptions, types of spore wall layers [21] and specialized AMF websites such as invam.wvu.edu, accessed on 9 May 2022.

The AMF assessment in the root samples followed the procedures of [22]. The complete root systems were cleared with 2.5% KOH (25 g KOH in 1000 mL water) by heating in an autoclave at 121 °C for 15 min and then rinsed with tap water. Phenolic substances were removed by adding alkaline hydrogen peroxide (60 mL of 28–30% NH_4_OH, 90 mL of 30% H_2_O_2_ and 840mL distilled water) and roots were left standing in a hood for one hour. Subsequently, the roots were rinsed with tap water, acidified with 1% HCl and left for 30 min. The HCl was decanted and without rinsing the roots, a staining reagent of 0.05% tryptan blue in acid glycerol (500 mL glycerol, 450 mL water), 50 mL of 1% HCl and 0.5 g tryptan blue) was added and roots were placed in an autoclave at 121 °C for 5 min. The stain was decanted and a de-staining solution comprising acid glycerol (500 mL glycerol, 450 mL water and 50 mL of 1% HCl) was added. Fine root segments were cut into approximately one cm-long pieces and 30 pieces were randomly picked, mounted on slides and observed under a compound microscope to assess the frequency and intensity of AMF colonization [23].

### 2.4. Greenhouse Experiment

The greenhouse experiments were laid out in a completely randomized design (CRD) consisting of seven treatments (six biological agents and water used as control). The biological agents included (1) Trichotech^®^ WP (Dudutech Ltd., Naivasha, Kenya), containing *Trichoderma asperellum* (4.0 × 10^9^ spores. g^−1^), (2) Bio-cure B^®^ (Bukoola Chemical Industries Ltd., Kampala, Uganda) containing *Pseudomonas fluorescens* (1 × 10^9^ cells. mL^−1^), (3) Rhizatech ^®^ (Dudutech Ltd., Naivasha, Kenya) that contains spores and mycelial fragments of Arbuscular Mycorrhizal Fungi (AMF) at 50 propagules per cm^3^. (4) *Trichoderma* isolate coded T10 and (5) *B. subtilis* isolate coded CA5 were obtained from the Plant pathology laboratory, Faculty of Agriculture, University of Nairobi and (6) Indigenous AMF were isolated as described above.

In vitro plantlets of clone 19,016 were planted in 300 mL pots with peat moss and hardened for 12 days. Then they were inoculated, following the manufacturers’ procedures, with Trichotech^®^, Bio-cure B^®^, or Rhizatech^®^. Cow manure was used as a carrier for *Bacillus subtilis* and *Trichoderma* isolates. Following guidelines by [24] with minor modification, 50 mL of colonized cow manure solution containing 1.5 × 10^13^ CFU. g^−1^ of *B. subtilis* and 4.71 × 10^8^ CFU. g^−1^ of *Trichoderma* were inoculated in a pot, respectively. For the native AMF, the previously described soil from the leek trap culture was used. This inoculum contained 400 spores of AMF per 100 g and was added at a ratio of 25 g inoculum per 250 g peat per pot. Half of the inoculum was applied to the center of the pot and the remaining amount was placed on top and covered with a layer of peat moss. Each treatment had 30 plantlets and the experiment was repeated three times. Watering was done in three-day intervals. After two months, the colonization of the roots was studied as well as plant height, number of leaves per plant, stem diameter, root diameter, root length, root collar diameter, number of roots per plant and biomass.

### 2.5. Field Experiment

Four weeks before field planting, four months old plantlets of clone 19,016 (30 plants per treatment) were inoculated with Trichotech^®^, Bio-cure B^®^, Rhizatech^®^, *Bacillus subtilis* isolate, *Trichoderma* isolate and indigenous AMF as described above. Control treatments comprised noninoculated in vitro plants and seedlings in Kiambere soil. Just before field planting, a limited amount of root tissue was used to assess root colonization. A Randomized Completely Block Design (RCBD) with all eight treatments was used for the field experiment. The seedlings were planted in November 2020 at Kiambere in pits prepared following the procedures by [25] with minor modifications. Every hole was 40 cm × 40 cm × 40 cm in size and during their preparation, the topsoil was separated from the subsoil. The distance between the plants was 3 m within and between the rows. An experimental plot consisted of ten plants arranged in two lines. After transplanting, the plants were watered twice a week (1.3 L per plant) for four months.

### 2.6. Data Collection and Statical Analysis

In the greenhouse experiment, data were collected two months after inoculation. The variables assessed were survival rate, shoot and roots variables. Shoot growth parameters included plant height (cm), number of leaves per plant and stem diameter (mm) and were assessed on 30 plants per repetition × 3 repetitions. Root parameters recorded were the number of roots per plant, root collar diameter (mm), root diameter (mm) and root length (cm). Destructive sampling of 10 plants was done to assess roots variables and plant biomass. Root diameter was collected at two centimeters from the root collar. The shoot and roots were oven-dried at 70 °C for 72 h before the shoot and root dry weight determination. The seedling quality index (DQI) was calculated according to [26], as shown in equation (Equation (1)).

(1) $$\mathrm{DQI}=\frac{\mathrm{Plant}\;\mathrm{Dry}\;\mathrm{weight}\;\left(g\right)}{\frac{\mathrm{Height}\;\left(\mathrm{cm}\right)}{\mathrm{Collar}\;\mathrm{diameter}\left(\mathrm{mm}\right)}+\frac{\mathrm{Shoot}\;\mathrm{dry}\;\mathrm{weight}\;\left(g\right)}{\mathrm{Root}\;\mathrm{dry}\;\mathrm{weight}\;\left(g\right)}}$$

Evaluation of root colonization of four plants previously inoculated with AMF followed the described procedures. For each of the three replicates, 30 root segments were analyzed per plant.

The growth of the plants in the field was assessed at two-month intervals until six months had passed since planting. The data collected included survival rate under field conditions, plant height (cm), diameter at one decimeter height (ddh (mm)) and the number of leaves per plant. The leaf area was determined two months after planting. Pictures of five fully expanded leaves per plant were used to determine leaf area by image analysis (ImageJ). Statical analysis of collected data was performed using IBM^®^ SPSS^®^ statistics (version 28). One-way Analysis of Variance (ANOVA) was used to test the significant differences among treatments and Duncan’s multiple range test (*p* < 0.05) was used to separate the means.

## 3. Results

### 3.1. Indigenous Arbuscular Mycorrhiza Fungi Identification and Spore Counting

**Figure 1:**
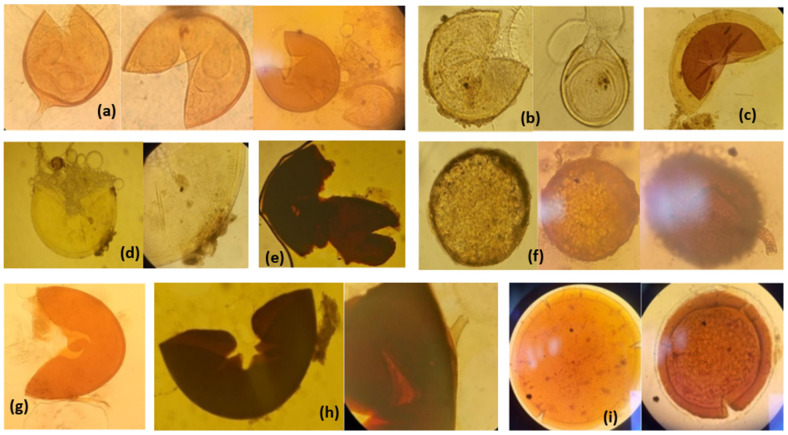
Different AMF spore morphotypes from the rhizosphere of *M. volkensii* seedlings in Kiambere soil. (**a**) Glomoid type of spore. (**b**) *Acaulospora scrobiculata* PVLG. (**c**) *A. scrobiculata* PVLG + Melzer. (**d**) *Gigasporaceae* PVLG. (**e**) *Gigasporaceae* PVLG + Melzer. (**f**) *Sporocarp* in the formative stage with thin mycelial sheath. (**g**) *Gigaspora margarita.* (**h**) *Gigasporaceae* PVLG + Melzers. (**i**) *Diversispora* sp. with expanding walls, spore at the center is parasitized and far-right is stained with Melzer’s reagent.

The average percentage of root colonization in the initial Kiambere nursery soil was 60%, whereas six spores per 100 g of soil were counted. In addition, there was an average occupancy of hyphae (96%), vesicles (52%), intraradical coils (9%) and appressoria (12%) (Supplement S1). The presence of appressoria obviously indicated that the soils still had active infectious AMF propagules. The trap culture with leek raised the number of spores to 400 per 100 g and 13 spore morphotypes in both Melzer’s and PVLG mounting reagents were identified (Figure 1 and Supplement S2). The identified morphotypes included six Glomoid, one Gigasporaceae (*Gigaspora margarita*) and two *Acaulosporaceae* species, one of them *Acaulospora scrobiculata*. Two sporocarpic forms and one *Diversispora* sp. could not be placed in any taxa.

### 3.2. Greenhouse Experiment

#### 3.2.1. Survival and Shoot Growth

Treating in vitro-grown *M. volkensii* plantlets with biologicals and native AMF significantly (*p* = 0.005) increased the survival rate compared to the control. Biological agents had an effective impact on plant height, number of leaves per plant and stem diameter (*p* < 0.001). The highest plant height and number of leaves per plant were recorded in plants treated with Rhizatech^®^, Native AMF, Bio-cure B^®^ and *Trichoderma* isolate (Table 3). A significantly wider stem was recorded in the treatments with Rhizatech^®^, Bio-cure B^®^ and native AMF (Table 3).

##### Root Growth and Fungal Colonization

**Figure 2:**
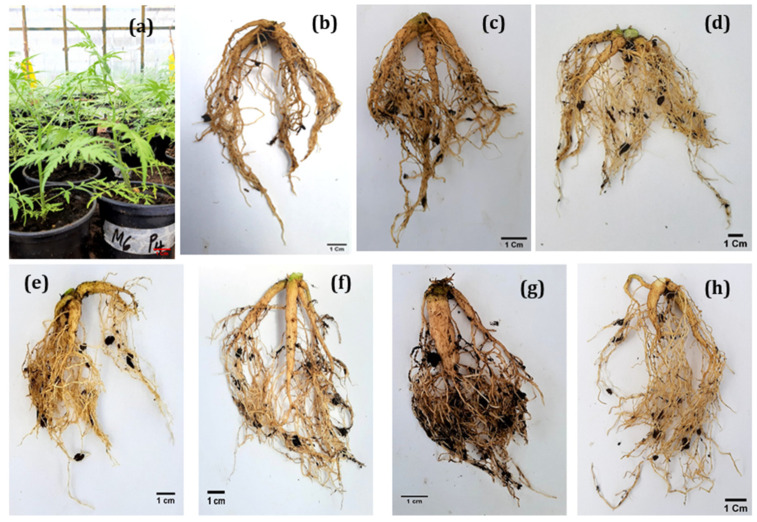
Root morphology of micropropagated *M. volkensii* during acclimatization in response to biological agents. (**a**) hardened plants under greenhouse conditions. (**b**) Control (noninoculated plant). (**c**) *Bacillus subtilis*. (**d**) Rhizatech^®^. (**e**) Trichotech^®^ WP. (**f**) Bio-cure B^®^. (**g**) *Trichoderma* isolate. (**h**) Indigenous AMF. Scale bar = 1 cm.

**Figure 3:**
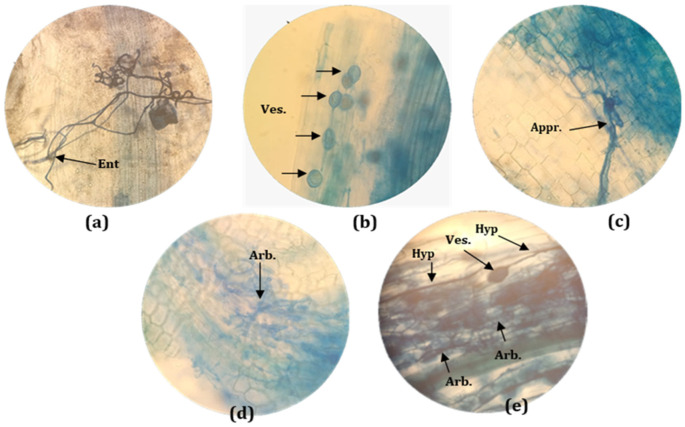
Different structures of AMF colonization in *M. volkensii* root segments view photos taken under a microscope at 400x magnification. (**a**) AMF entry point. (**b**) root segments with multiple AMF vesicles. (**c**) AMF appressoria. (**d**) root segments with multiple arbuscules. (**e**) Roots segment with hyphae, vesicles and arbuscules. Ent: entry point. Ves: Vesicles. Appr.: appressoria. Arb.: arbuscules. Hyp: Hyphae.

**Figure 4:**
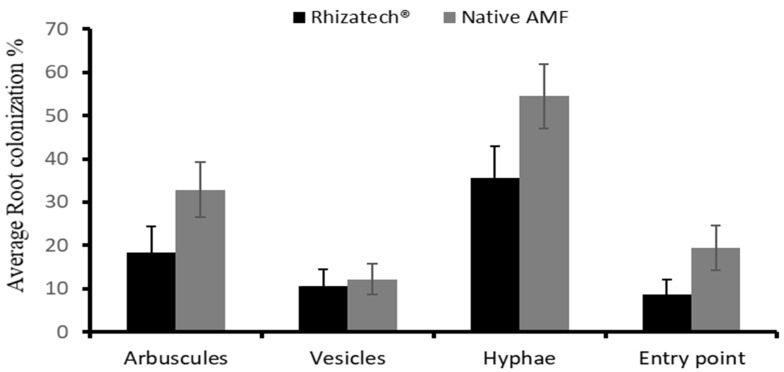
*Melia volkensii* root colonization percentages of two AMF two months after inoculation. Histograms represent means ± SE from 12 plants and 30 root segments were analyzed per plant.

The biological agents had a significant impact on the root growth parameters after two months of acclimatization (Table 4). The root diameter of the plants treated with Rhizatech^®^ and Bio-cure B^®^ was larger than in the other treatments. In general, all inoculated plants had longer roots than the control treatment, but the plants inoculated with the native AMF had the longest roots (13.5 cm). Rhizatech^®^ (7.1 mm), Bio-cure B^®^ (7 mm) and *B. subtilis* (7 mm) induced the largest root collar diameter. The number of roots per plant did not differ significantly between the inoculated plants and the control. However, the inoculated plants generally developed multiple secondary roots (Figure 2). After two months, AMF had clearly colonized the acclimatized plantlets. Entry points, appressoria, hyphae, vesicles and arbuscules were observed on stained roots under a microscope at 40× magnification (Figure 3). Two months after AMF inoculation, the formation of hyphae was observed in 54.5% of the roots and arbuscules were observed in 32.9% (Figure 4). In contrast,12.2% and 19.4% of observed roots displayed vesicles and entry points, respectively. Native AMF showed a better root colonization rate than AMF-based product (Rhizatech^®^).

##### Effect of the Biological Agents on Quality and Biomass

All biologicals significantly (*p* < 0.001) increased the DQI and the fresh and dry weight of the root and shoot (Table 5). DQI ranged from 0.39 to 0.52 for all biologicals, while only 0.21 was obtained for the control treatment. The highest fresh and dry weight of shoots was recorded in plants treated with Bio-cure B^®^ and Rhizatech^®^. The root and shoot parameters of the plants inoculated with native AMF exceeded those of the control treatment but not those of the other treatments. 

### 3.3. Field Experiment

**Figure 5:**
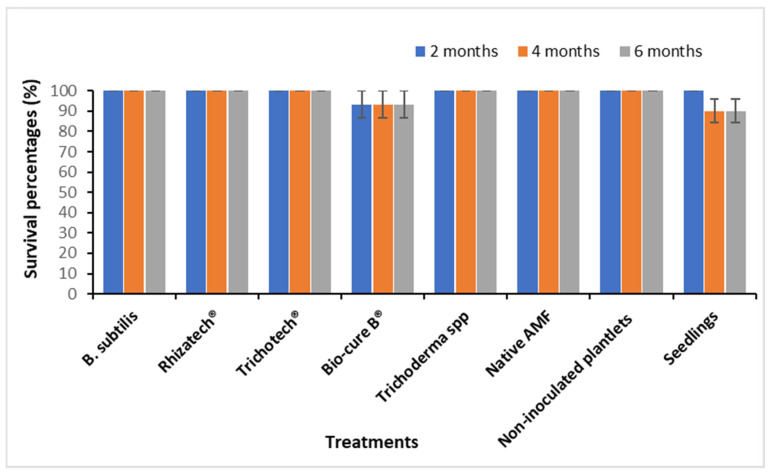
The field survival rate of *M. volkensii* plants under Kiambere semi-arid conditions two, three and six months after planting.

**Figure 6:**
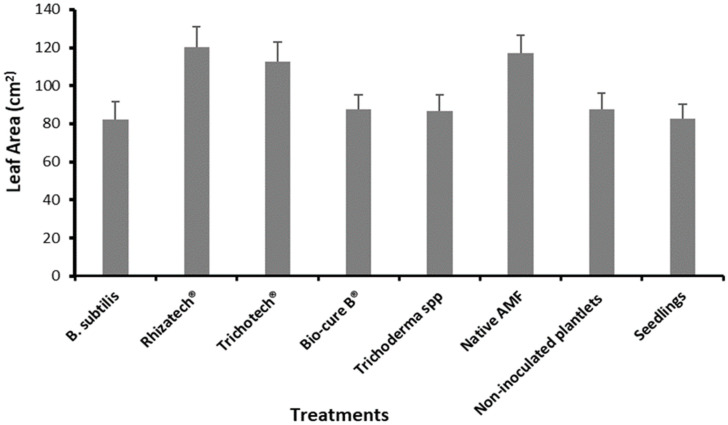
Leaf area (cm^2^) as affected by biological agents during field establishment two months after planting *M. volkensii* under Kiambere semi-arid conditions.

**Figure 7:**
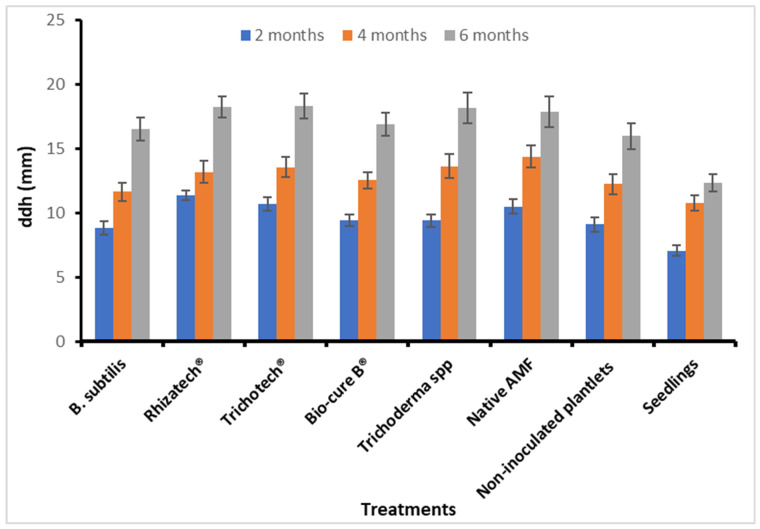
Diameter at one decimeter height (ddh) as affected by biological agents after two, three and six months of planting *M. volkensii* under Kiambere semi-arid conditions.

A root colonization percentage by AMF of 25% was recorded just before planting and AMF entry point, appressoria, hyphae, vesicles and arbuscules were also observed in roots segments. After six months under field conditions, micropropagated *M. volkensii* had a survival rate ranging from 93% to 100%, while conventional seedlings had a survival rate of 90 %. The biological agents showed no significant difference in survival rate (Figure 5) but did cause a significantly larger leaf area after two months, with the largest leaves recorded with Rhizatech^®^, native AMF and Trichotech^®^ (Figure 6). The largest ddh was recorded in plants treated with Rhizatech^®^, Trichotech^®^ and Native AMF (Figure 7). As the plants grew, the difference in ddh between in vitro plants and conventional seedlings increased. 

**Figure 8:**
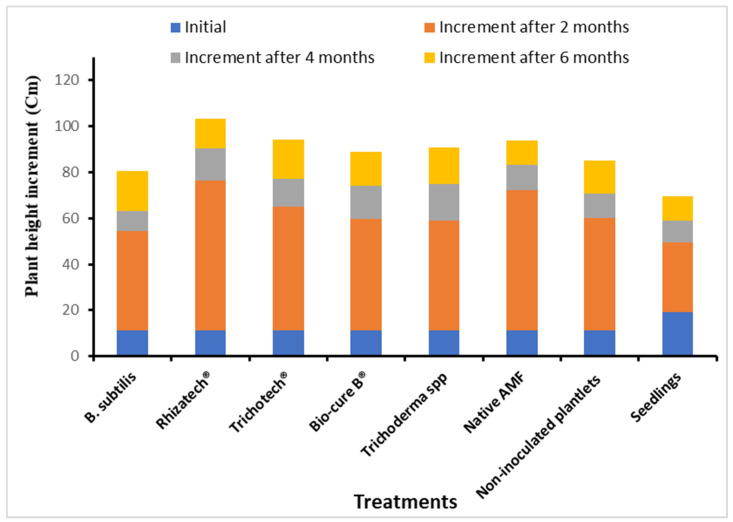
*Melia volkensii* plant height increment as enhanced by biological agents after two, four and six months in Kiambere semi-arid conditions.

Two months after transplanting, we also observed quite some differences in the increase of plant height between the treatments. Plants inoculated with Rhizatech^®^ and Native AMF grew faster; their length increased by 65.1 cm and 61.0 cm, respectively. In contrast, the weakest increase in plant height was observed in the non-inoculated seedlings (Figure 8). The growth differences between the inoculated plants were levelled out between two and four months and between four and six months, except for those inoculated with *B. subtilis*. After six months, the plants inoculated with Rhizatech^®^, Trichotech^®^, Native AMF, Trichoderma and Bio-cure B^®^ reached the highest plant length (Figure 8).

**Figure 9:**
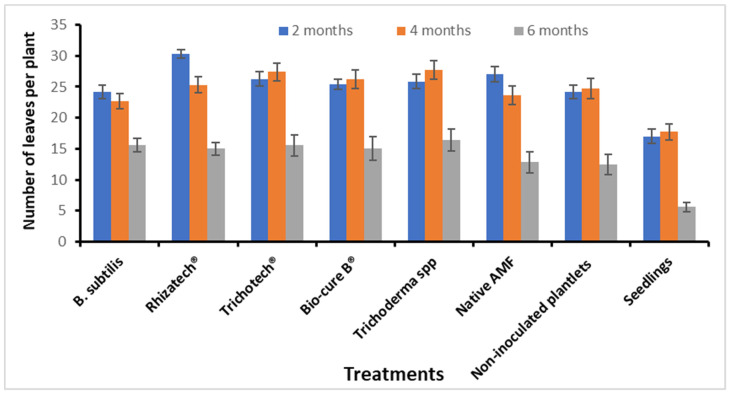
The number of leaves per plant as affected by biological agents after two, four and six months of planting *M. volkensii* under Kiambere semi-arid conditions.

**Figure 10:**
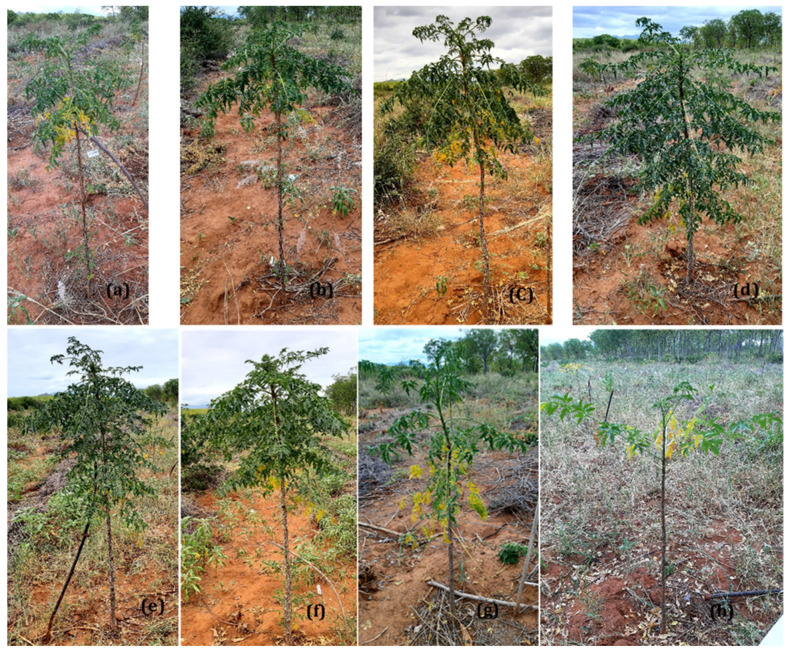
*Melia volkensii* plant’s response to different biological agents six months after transplanting under semi-arid conditions in Kiambere. (**a**) Plants treated with *Bacillus subtilis*. (**b**) Plants treated with Rhizatech^®^. (**c**) Plants treated with Trichotech^®^ WP. (**d**) Bio-cure B^®^. (**e**) *Trichoderma* isolate. (**f**) Indigenous AMF. (**g**) non-inoculated in vitro plants. (**h**) Plants conventionally propagated through seed.

Two months after planting, the number of leaves was significantly higher in plants treated with Rhizatech^®^, whereas the lowest leaf number was observed in conventional seedlings. After four months, no significant difference was observed anymore in the number of leaves between Rhizatech^®^, Trichotech^®^, Bio-cure B^®^, *Trichoderma,* Native AMF and water control (Figure 9). Four months after transplanting, the lack of rain took its toll and the leaves started to fall off. The degree of leaf fall differed between treatments. The plant inoculated with *B. subtillis*, Rhizatech^®^, and native AMF showed a slight loss of leaves. Although after six months, the conventional seedlings lost the most leaves than other treatments (Figure 10). No significant differences were recorded between plants treated with biological agents and in vitro plants.

## 4. Discussion

### 4.1. Greenhouse Experiment 

A successful transfer of *M. volkensii* plants from in vitro to the greenhouse, followed by the establishment in the field, is crucial for any cloned tree planting strategy. This study illustrates that inoculating the in vitro-grown plantlets with biological agents increased the survival of the plantlets to 100%. Similar findings have been reported in micropropagated *Persea americana* [10], *Castanea sativa* [27], *Quercus suber* L. [28] and *Citrus tangerine* [13], where ectomycorrhizal fungi increased the survival rate over non-inoculated plants during acclimatization. This could be attributed to the inhibition of rhizosphere pathogens by beneficial microorganisms such as *Trichoderma* spp., *B. subtilis*, AMF and *P. fluorescens*. Pandey et al. [11] reported that *B. subtillis* and *P. corrugata* suppressed root pathogens such as *Fusarium oxysporum* during tea plantlet acclimatization. Root rot and wilting were prevented during acclimatization by inoculating tea plantlets with *P. fluorescens*, *Azospirillum brasilense* and *T. harzianum* [29]. The authors suggested that the rise in peroxidase and phenylalanine ammonia lyase activities in inoculated plants indicate the onset of defense mechanisms. *Azospirillum brasilense Sp245* protected *Prunus cerasifera L.* plantlets against *Rhizoctonia spp* [30]. All biological agents used in this study yielded similar survival rates, consistent with the results reported by [11] in tea hardening. At the nursery stage, *Trichoderma*, AMF and *Bacillus* improved the survival of micropropagated bananas [14].

During acclimatization, the inoculated microorganisms improved *M. volkensii* shoot growth (plant height, number of leaves per plant, stem diameter) and root growth (root length, root diameter and root collar diameter). The effects on shoot growth varied among microorganisms. Especially Rhizatech^®^, local AMF and Bio-cure B^®^ (*P. fluorescens)* significantly increased plant height, number of leaves per plant and stem diameter. Similar findings were reported by [27], who evaluated the effect of four ectomycorrhizal fungi in *C. sativa*. Similarly, *Eucalyptus tereticornis* plants inoculated with local isolates of mycorrhizal fungi, including *Pisolithus tinctorius,* displayed superior growth compared with non-mycorrhizal plants. The inoculation of tea plantlets with *B. subtillis* and *P. corrugate* promoted shoot growth [11]. *Pinus patula* seedlings treated with Rhizatech^®^ were superior in plant height [31]. Similar findings were reported by [14], who revealed that Rhizatech^®^, *Bacillus* and *Trichoderma* promoted the development of banana plants. The increased growth observed in *M. volkensii* treated with microorganisms could be attributed to the promotion of nutrient absorption by *T. harzianum* [32], *T. asperellum* [33], *P. fluorescens* [34] and AMF [35,36]. Thomas et al. [29] reported that the occurrence of beneficial microorganisms during acclimatization facilitated nutrient uptake by tea plants. 

Pandilla et al. [37] revealed that AMF induced different root morphology of *Pouteria lucuma*. In vitro mycorrhization of pear plants improved root architecture and nutrient composition after acclimatization [38]. An increase in root diameter during the hardening of *Prunus dulcis* has also been reported when inoculated with endomycorrhizas such as *G. mosseae* and *S. calospora* [39]. This study showed no significant differences in the number of roots between all treatments, which was similar in tea plants [29] and pears [38]. Similar results were also reported in *Prunus persica* × *P. amigdalus* (GF 677)*, Prunus cerasifera* × *P. spinosa* (Mr.S 2/5 plum) and Northen Spy × M1 (MM 106 apple) rootstocks treated with *A. brasilense* Sp245 [40]. Halifu et al. [41] found that *Trichoderma* increased plant height and significantly boosted the number of root tips, root diameter, root length and root area of *Pinus sylvestris* var. *mongolica* seedlings. Furthermore, *Pseudomonas* and *Bacillus* promoted the root development of conifers [42]. 

The DQI estimated that the biological agents increased the quality of micropropagated *M. volkensii* plants by about 50% under greenhouse conditions. Our results agree with [43], who reported that *A. scrobiculata* and *Rhizophagus irregularis* improved the quality of *Aegle marmelos*, *Leucaena leucocephala* and *Parkia roxburghii* seedlings. This can be attributed to the positive effect of AMF on plant adaptability to external conditions [44] and improved tolerance to diseases [45]. *Trichoderma virens* and *T. harzianum* provided the most significant increases in DQI of *E. camaldulensis* [46].

We showed that root and shoot fresh and dry weight significantly increased after inoculating with microorganisms. *Bacillus subtillis*, at 2 × 10^7^ UFC/mL, applied in the substrate (vermiculite, pine bark and NPK) increased the Dickson quality index and dry biomass of *Pinus taeda* seedlings [47]. Martins et al. [27] showed that ectomycorrhizal fungi significantly increased the fresh weight of micropropagated *C. sativa*. *Pseudomonas fluorescens*, *Azospirillum brasilense* and *T. harzianum* increased the fresh weight of in vitro derived tea plants under acclimatization [29]. The positive effects of AMF on dry weight agree with [10], who reported *G. fasciculatum* significantly increased root and shoot dry weight of tissue cultured *P. americana* plants. Similar findings were reported by [13], who noted that *G. mosseae* enhanced citrus shoot and dry weight. The increase in plant biomass can be attributed to improved water relations when microorganisms were inoculated to in vitro-raised plants [48]. This can also be credited to the ability of AMFs to improve plant growth, nutrient uptake, stress tolerance and disease resistance [49]. Photosynthesis rates, transpiration rates and stomatal conductance increased when AMF were inoculated in citrus [13], which improved the plants’ quality, biomass and growth during growth acclimatization. *Pseudomonas fluorescens* improved cell elasticity, water stress tolerance and growth in *Pinus halepensis* seedlings [50]. The authors reported that combining the ectomycorrhizal fungus *P. fluorescens* with *P. tinctorius* caused an improvement in osmotic regulation.

### 4.2. Field Experiment

Before transplanting, typical AMF structures such as appressoria, arbuscules, hyphae and vesicles were observed. The development of arbuscles suggested an intimate nutrient exchange between the fungus and the young melia trees.

Two, four and six months after transplanting under Kiambere semi-arid conditions, the survival rate showed no significant differences and it ranged from 90% in conventional seedlings to 100% in seedlings inoculated with Trichotech^®^, Rhizatech^®^, *B. subtilis* and *Trichoderma* isolate or native arbuscular mycorrhizal fungi. The high survival rate recorded could be due to the protective effect of biological agents [11,30] and their regulation of biotic and abiotic stresses [51].

In the first two months after transplanting, plants treated with AMF significantly developed more leaves and diameter at one decimeter height (ddh), leaf area and plant height significantly increased compared to the other treatments. Sakha et al. [52] observed that inoculating sweet potatoes with AMF elicited many branches and vine length. This is probably due to the rapid establishment of the root system of AMF-treated plants under semi-arid conditions. The enhanced root system allowed the plants to absorb nutrients more efficiently [15]. Dominguez et al. [53] stated that five months after inoculating *P. halepensis* seedlings with *P. fluorescens* and *Tuber melanosporum*, nutrient uptake and growth were improved. *Pseudomonas* spp. and *T. rifaii* treatments significantly increased plant height and aerial parts of *Hibiscus sabdariffa* L. [54]. The native AMF used in the current study originated from Kiambere and therefore adapted well to the same climatic conditions more quickly than other microorganisms. In addition, the highest increment in plant height was recorded in the first two months, which could be attributed to the combined effect of biological agents used and conducive rainfall and watering twice per week up to four months. After four months and six months, the inoculated plants did not grow faster than the non-inoculated ones. This implies that inoculation with microorganisms is particularly beneficial for the early development of *M. volkensii* in the field under semi-arid conditions.

## 5. Conclusions

We investigated for the first time the effect of inoculation with symbiotic bacteria and AMF on the survival and growth of in vitro propagated *M. volkensii*. They increased survival and plant quality during the first two months and stimulated growth. The in vitro plantlets were successfully planted under semi-arid conditions and grew better than conventional seedlings. Field performance was enhanced by our own native AMF inoculum but also by commercial mixtures such as Rhizatech^®^, Bio-cure B^®^, *Trichoderma* isolate and Trichotech^®^. Integrating bacteria and AMF during the establishment process of micropropagated *M. volkensii* could solve poor plant growth and establishment in semi-arid conditions. Further research into the interaction of these microbes with the roots of *M. volkensii* in different arid and semi-arid regions is recommended.

## Figures and Tables

**Table 1:** Monthly rainfall and temperature information during field experiment period November 2020 to May 2021.

Month	Rainfall (mm)	Minimum Temperature (°C) *	Maximum Temperature (°C) *
November 2020	252	13.23	24.57
December 2020	83	11.84	25.29
January 2021	31.5	11.58	26.03
February 2021	29.3	12.61	26.18
March 2021	0	13.13	26.58
April 2021	96.3	15.17	25.37
May 2021	0	14.52	21.52

* Adapted from http//www.accuweather.com accessed on 20 September 2021.

**Table 2:** Soil chemical and physical properties in field site at Kiambere before planting.

Property	Units	Value	Property	Units	Value
pH	-	6.35	Iron (Fe)	ppm	48.9
Electrical conductivity (EC)	dS/m	2.1	Manganese (Mn)	ppm	66.5
Organic carbon (OC)	%	0.85	Copper (Cu)	ppm	Trace
Cation exchange capacity (CEC)	Cmol/kg	10	Calcium (Ca)	Cmol/kg	7.8
Nitrogen (N)	%	0.12	Sulfur (S)	ppm	2.8
Phosphorus (P)	ppm	8.43	Calcium (Ca)	Cmol/kg	3.1
Potassium (K)	Cmol/kg	0.55	Sand	%	60
Sodium (Na)	Cmol/kg	0.55	Silt	%	2
Zinc (Zn)	ppm	0.79	Clay	%	38
Magnesium (Mg)	Cmol/kg	2.01	Texture		Sandy clay
Boron (Bo)	ppm	1.5			

**Table 3:** Plantlets survival rate and shoot growth of *M. volkensii* plants after two months under acclimatization.

Treatments	Survival Rate (%)	Plant Height (cm)	Number of Leaves per Plant	Stem Diameter (mm)
*B. subtilis*	98.0 ± 1.11 a	6.4 ± 0.27 d	8.9 ± 0.27 b	3.4 ± 0.10 c
Rhizatech^®^	100.0 ± 0.00 a	9.3 ± 0.43 a	10.2 ± 0.28 a	4.0 ± 0.12 a
Trichotech^®^	99.0 ± 1.11 a	7.0 ± 0.44 d	8.9 ± 0.37 b	3.5 ± 0.11 bc
Bio-cure B^®^	96.7 ± 1.92 a	8.5 ± 0.48 ab	9.9 ± 0.38 a	3.9 ± 0.12 a
*Trichoderma*	100.0 ± 0.00 a	7.6 ± 0.38 abc	10.1 ± 0.29 a	3.6 ± 0.11 bc
Native AMF	100.0 ± 0.00 a	8.8 ± 041 ab	9.8 ± 0.25 a	3.8 ± 0.10 ab
Control (Water)	89.7 ± 0.33 b	6.3 ± 0.26 d	8.6 ± 0.23 b	3.4 ± 0.09 c
Mean	97.6	7.7	9.5	3.7
*p*-value	0.005	<0.001	<0.001	<0.001

Means followed by similar letters within a column are not significantly different at *p* ≤ 0.05. Duncan’s multiple range test at 5% was used to separate means. Data are reported as mean ± standard errors.

**Table 4:** *Melia volkensii* root growth as affected by different biological agents during acclimatization after two months.

Treatments	Root Diameter (mm)	Root Length (cm)	Root Collar Diameter (mm)	Number of Roots
*B. subtilis*	5.6 ± 0.35 ab	13.1 ± 0.45 a	7.0 ± 0.18 a	4.9 ± 0.53
Rhizatech^®^	6.5 ± 0.28 a	13.2 ± 0.58 a	7.1 ± 0.21 a	4.6 ± 0.41
Trichotech^®^	5.5 ± 0.39 ab	12.2 ± 0.48 a	6.2 ± 0.21 b	4.6 ± 0.48
Bio-cure B^®^	6.22 ± 0.33 a	12.8 ± 0.44 a	7.0 ± 0.17 a	4.0 ± 0.33
*Trichoderma*	5.6 ± 0.31 ab	12.4 ± 0.56 a	6.8 ± 0.16 ab	5.8 ± 0.60
Native AMF	6.0 ± 0.24 ab	13.5 ± 0.51 a	6.7 ± 0.17 ab	4.9 ± 0.44
Control	5.1 ± 0.32 ab	10.1 ± 0.54 b	6.7 ± 0.15 ab	5.0 ± 0.39
Mean	5.8	12.5	6.8	4.8
*p* value	0.035	<0.001	0.018	0.238

Means followed by similar letters within a column are not significantly different at *p* ≤ 0.05. Duncan’s multiple range test at 5% was used to separate means. Data are reported as mean ± standard errors.

**Table 5:** Quality and biomass of *M. volkensii* plantlets as affected by different by biological agents during acclimatization after two months.

Treatments	DQI	RFW (g)	SFW (g)	RDW (g)	SDW (g)
*B. subtilis*	0.4 ± 0.05 a	2.2 ± 0.19 a	2.4 ± 0.18 de	0.4 ± 0.04 a	0.6 ± 0.06 cd
Rhizatech^®^	0.5 ± 0.04 a	3.0 ± 0.14 a	4.3 ± 0.35 a	0.5 ± 0.03 a	1.0 ± 0.11 a
Trichotech^®^	0.4 ± 0.05 a	2.8 ± 0.51 a	2.7 ± 0.30 cd	0.4 ± 0.04 a	0.7 ± 0.08 bc
Bio-cure B^®^	0.5 ± 0.06 a	3.0 ± 0.20 a	3.7 ± 0.34 ab	0.5 ± 0.04 a	0.9 ± 0.09 ab
*Trichoderma*	0.4 ± 0.03 a	2.6 ± 0.18 a	3.5 ± 0.32 bc	0.4 ± 0.03 a	0.8 ± 0.06 bc
Native AMF	0.4 ± 0.06 a	2.4 ± 0.19 a	3.0 ± 0.25 bcd	0.4 ± 0.04 a	0.7 ± 0.07 bc
Control	0.2 ± 0.02 b	1.3 ± 0.11 b	1.7 ± 0.15 e	0.2 ± 0.02 b	0.4 ± 0.04 d
Mean	0.41	2.46	3.04	0.40	0.73
*p* value	<0.001	<0.001	<0.001	<0.001	<0.001

DQI: Dickson quality index. RFW: Root fresh weight (g). SFW: Shoot fresh weight (g). RDW: Root dry weight (g). SDW: Shoot dry weight (g). Means followed by similar letters within a column are not significantly different at *p* ≤ 0.05. Duncan’s multiple range test at 5% was used to separate means.

## Data Availability

The raw data used to support the findings of this study will be made available without undue reservation.

## Supplementary Materials

The following supporting information can be downloaded at: https://www.mdpi.com/article/10.3390/plants11101300/s1. Supplement S1: Colonization in potted *M. volkensii* seedlings from Kiambere: Pictures representing different forms of entry points with the hyphae at the point of penetrating the surface of the roots. Supplement S2: Different Arbuscular Mycorrhiza Spore Morphotypes potted *M. volkensii* seedlings from Kiambere.
