# Supplementary material for: Early Growth Performance of In Vitro Raised Melia volkensii Gürke Plantlets in Response to Beneficial Microorganisms under Semi-Arid Conditions
Source: Plants (Basel). 2022 May 13;11(10):1300. doi: 10.3390/plants11101300 (PMC9145849; doi:10.3390/plants11101300)
Supplement: Supplementary file 1 [file plants-11-01300-s001.zip › plants-1712217-supplementary.pdf]

Supplement S1. Colonization in potted *M. volkensii* seedlings from Kiambere: Pictures representing different forms of entry points with the hyphae at the point of penetrating the surface of the roots.

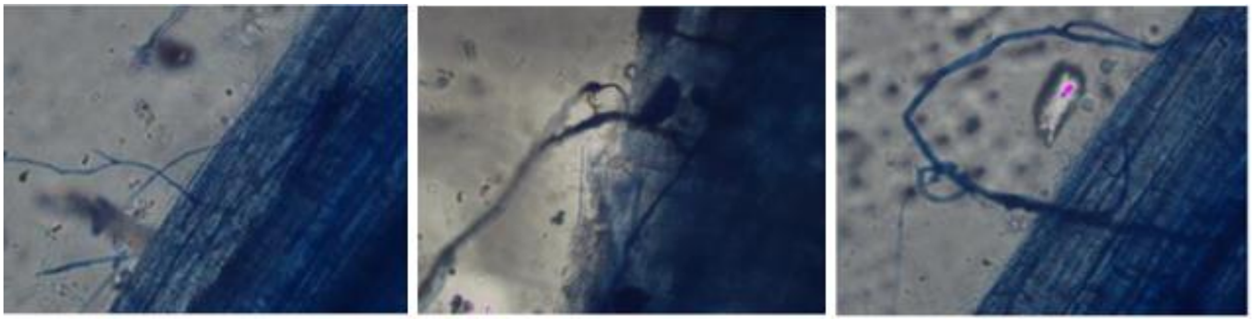

Supplement S2. Different Arbuscular Mycorrhiza Spore Morphotypes potted *M. volkensii* seedlings from Kiambere

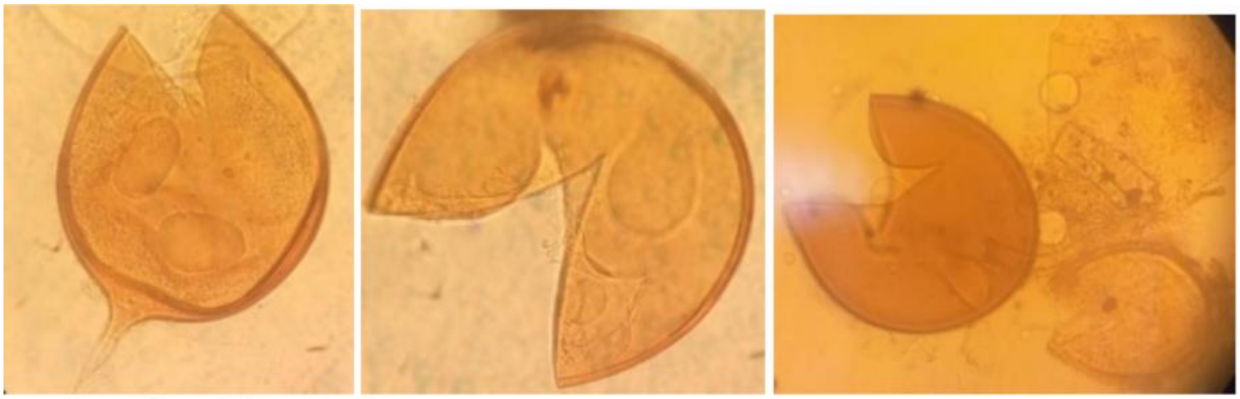

1. Glomoid type of spores

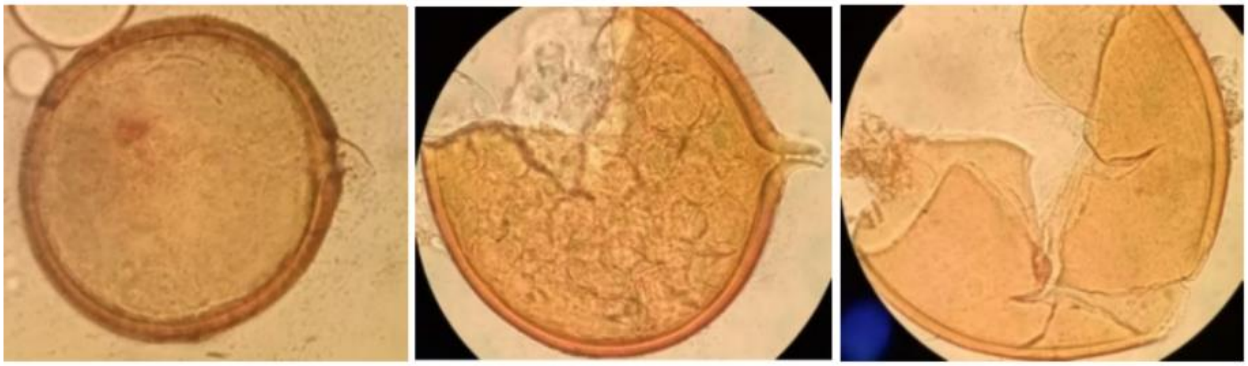

2. Glomoid spore type

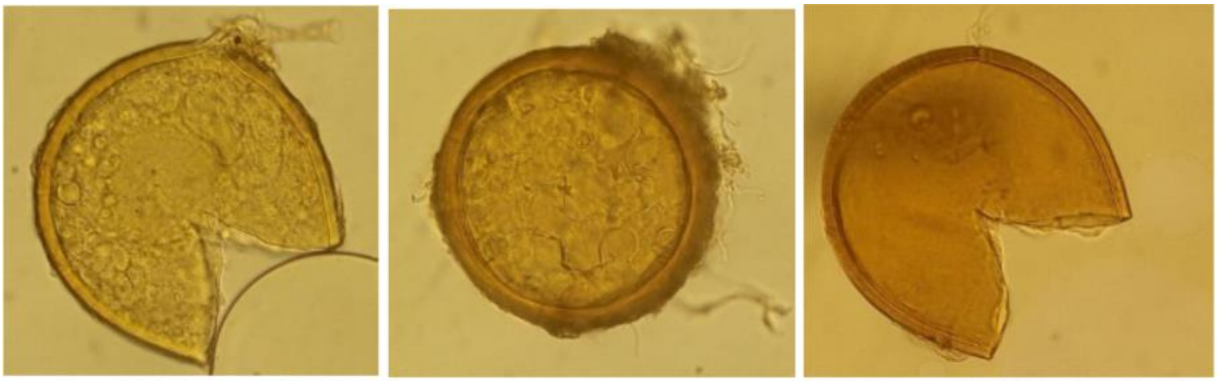

3. M1 Glomoid PVLG M1Glomoid PVLG + Melzer

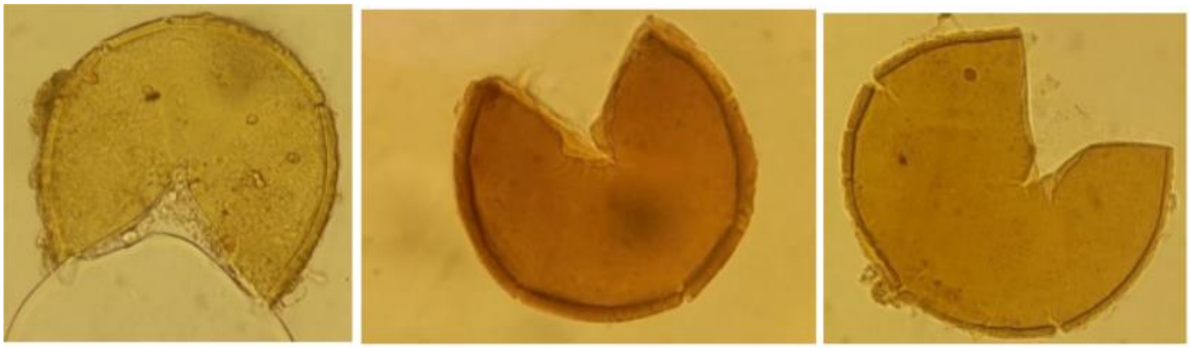

4. Glomoid PVLG M1 Glomoid PVLG+ Melzer

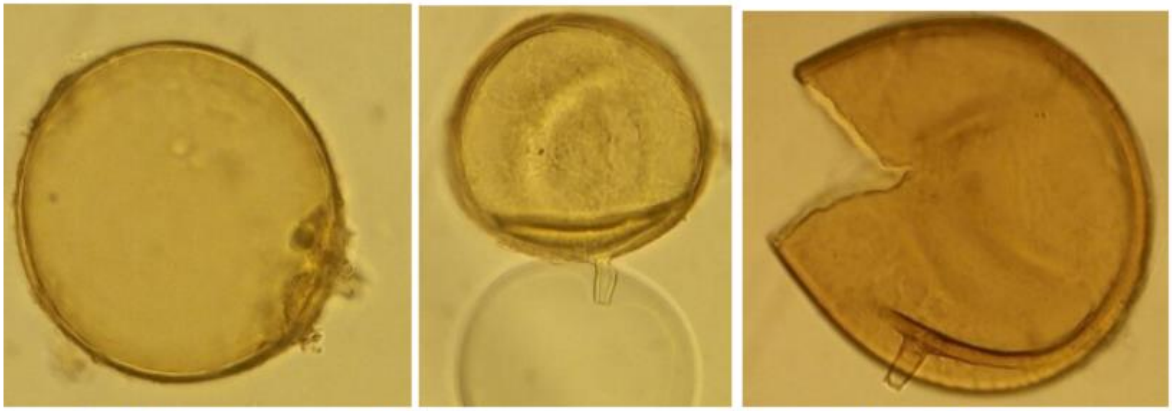

5. N91Glomoid PVLG N91 Glomoid PVLG + Melzer

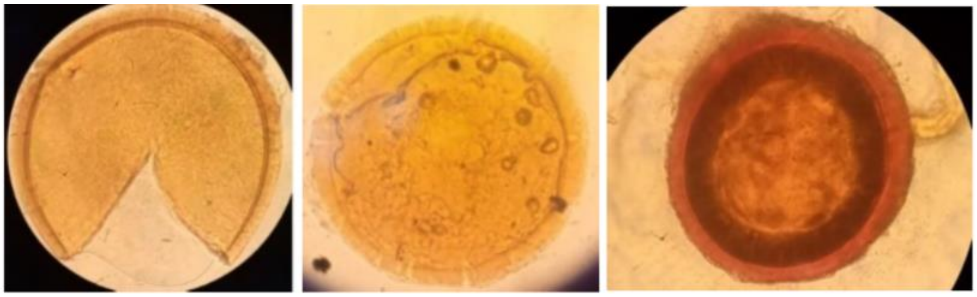

6. *Diversispora sp.* (With expanding walls), spore at center is parasitized and far right is stained with Melzers reagent.

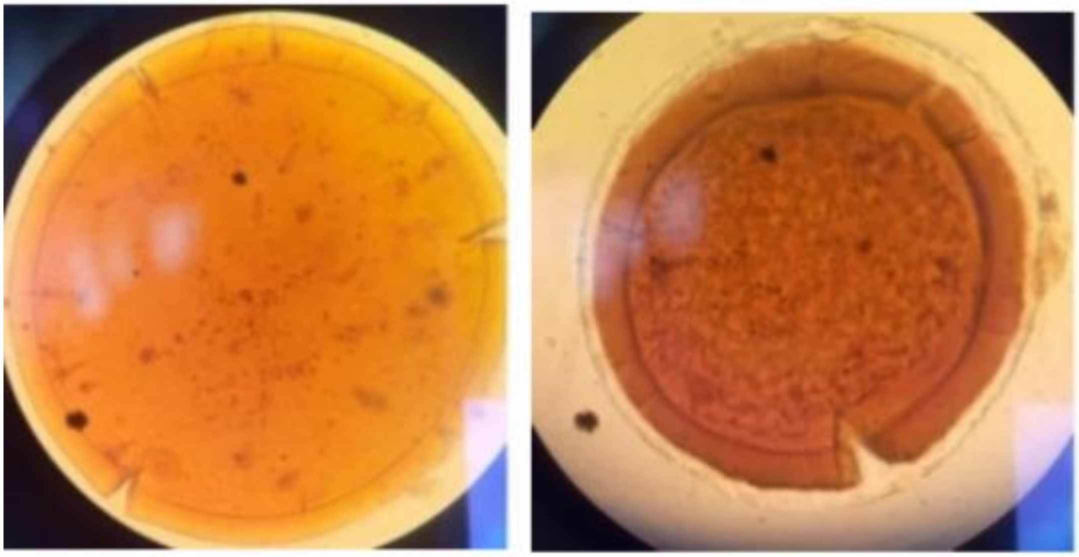

6. *Diversispora?* (Explanation as above)

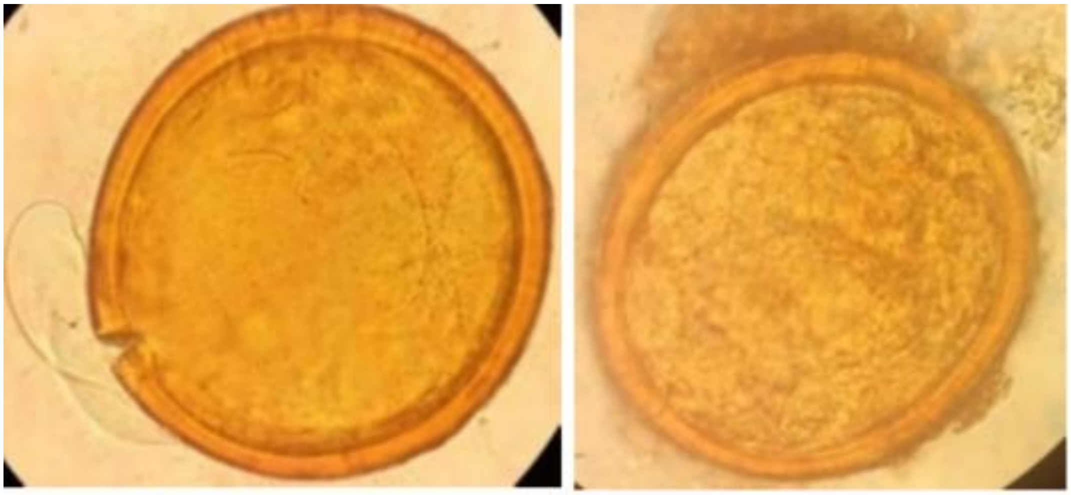

7. Glomoid species

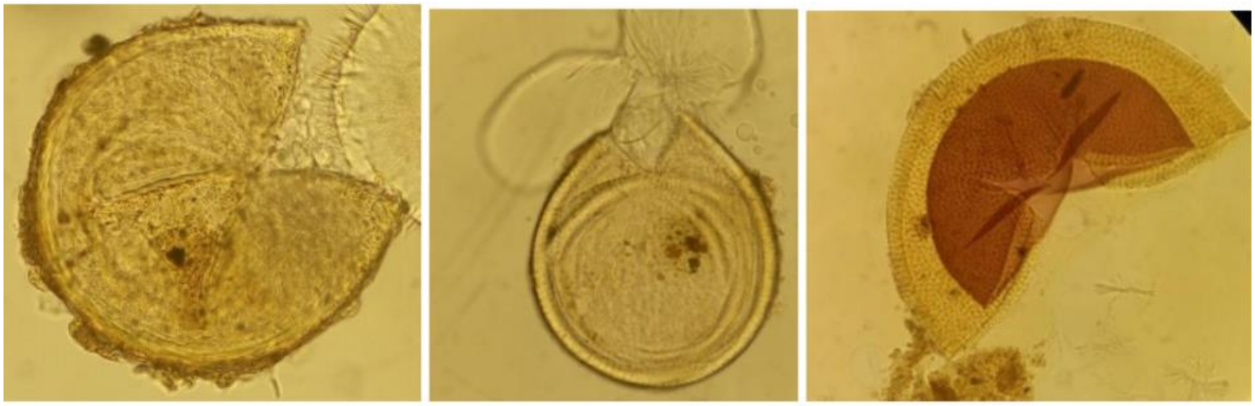

8. *Acaulospora scrobiculata* PVLG PVLG + Melzer

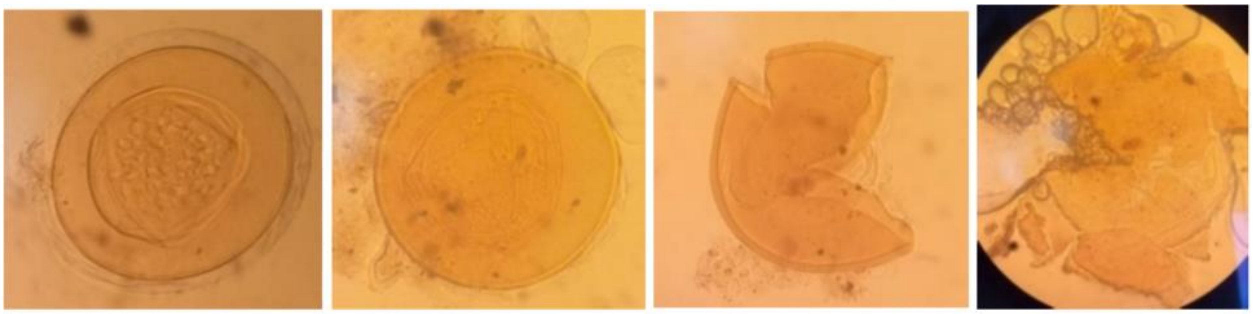

9. Acaulosporaceae 9. Acaulosporaceae

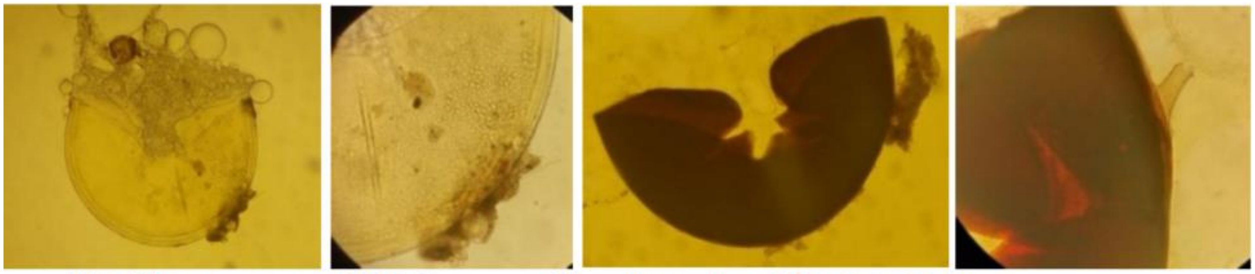

*10. Gigaspora*ceae PVLG *Gigasporaceae* PVLG + Melzers

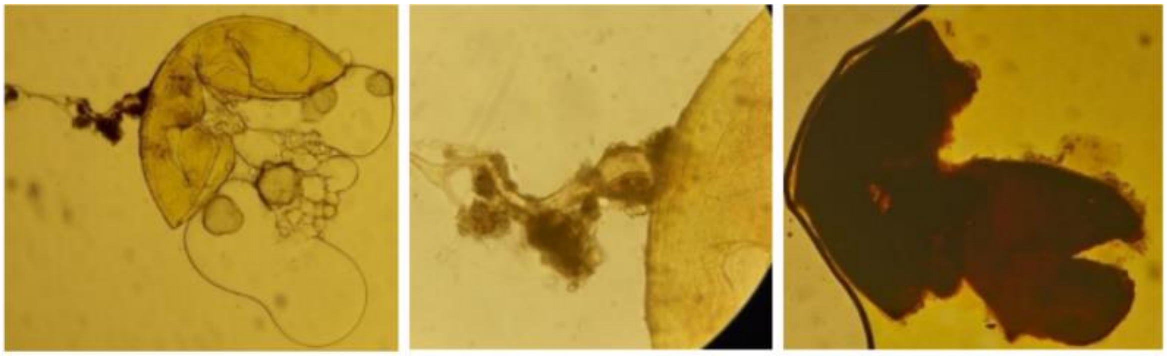

10. Gigasporaceae in PVLG Gigasporaceae PVLG + Mel

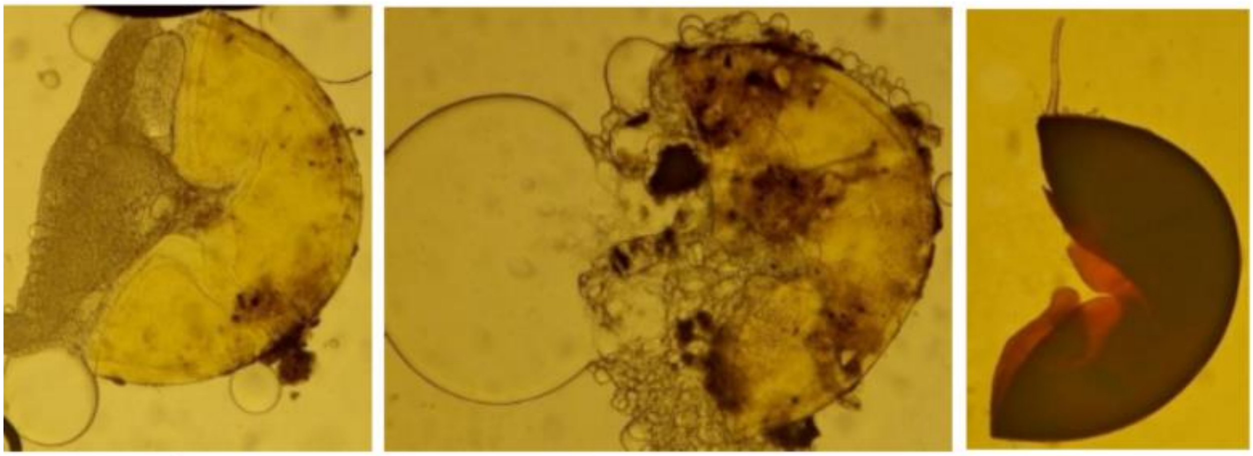

10. Gigasporaceae

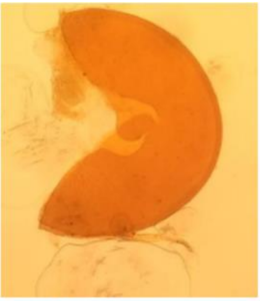

11. Gigaspora margarita

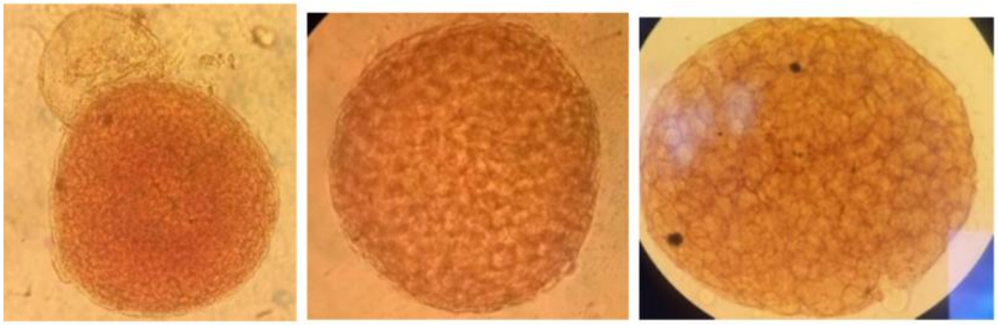

12. Uncertain Identity morphotype with defined shape and inner oily contents and ornamentation is consistent.

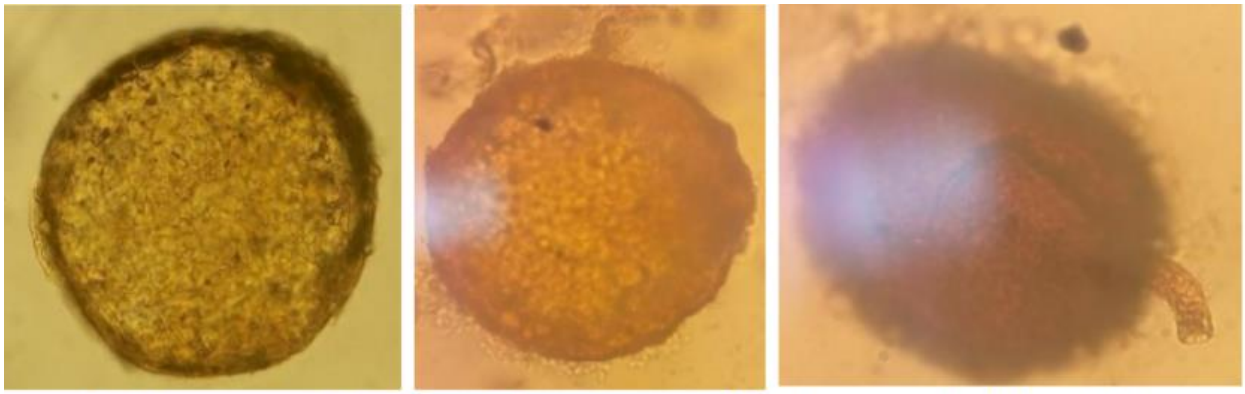

13. Sporocarp in formative stage with thin mycelial sheath?
